# Combination of oral fluoropyrimidines and EGFR-directed antibodies in frail patients with metastatic colorectal cancer: a systematic review

**DOI:** 10.1007/s00432-026-06639-x

**Published:** 2026-10-20

**Authors:** Alena Annbalou Hasenburg, Sebastian Stintzing, Arndt Stahler

**Affiliations:** 1 Department of Hematology, Oncology and Cancer Immunology (CCM), Charité - Universitaetsmedizin Berlin, Corporate Member of Freie Universitaet Berlin and Humboldt-Universitaet zu Berlin, Charitéplatz 1, 10117 Berlin, Germany; 2 German Cancer Consortium (DKTK), Partner Site Berlin, German Cancer Research Centre (DKFZ), Heidelberg, Germany

**Keywords:** Oral fluoropyrimidine, EGFR-antibodies, Metastatic colorectal cancer, Frail

## Abstract

**Background:**

Evidence of treatment strategies in frail patients (i.e. not fit for combination therapies) with unresectable metastatic colorectal cancer (mCRC) is limited to dose-reduced doublet regimens or oral fluoropyrimidines (FP) with bevacizumab. We conducted a systematic review analyzing evidence for oral FPs with epidermal growth factor receptor (EGFR)-directed monoclonal antibodies for patients with *RAS* wild-type (WT) mCRC across treatment lines.

**Methods:**

We searched PubMed and Cochrane Library for phase II and phase III studies evaluating oral FPs (capecitabine, S-1 or trifluridine/tipiracil (FTD/TPI)) plus EGFR-directed monoclonal antibody (panitumumab or cetuximab) in mCRC. Outcomes included efficacy and safety.

**Results:**

Seven publications met inclusion criteria (n = 262 patients). First-line data was limited to elderly cohorts receiving capecitabine plus cetuximab or panitumumab with a median progression-free survival (PFS)/median overall survival (OS) of 7.2/17.1 months (cetuximab) and 7.5/23.7 months (panitumumab), respectively. In third-line and beyond, median PFS for the combination of FTD/TPI plus cetuximab or panitumumab ranged from 2.4 (cetuximab) to 5.8 months (panitumumab), and OS from 9.8 (cetuximab) to 14.2 months (panitumumab). S-1 plus cetuximab was associated with a median PFS of 5.5 months and OS of 13.6 months.

**Conclusion:**

While capecitabine should not be used together with anti-EGFR antibodies, FTD/TPI in combination with an EGFR-directed antibody appears feasible, shows promising activity and manageable toxicity profile in RAS WT mCRC potentially offering an alternative for frail patients; prospective trials are needed.

## Introduction

Although frail patients represent a large proportion of patients with unresectable metastatic colorectal cancer, they are underrepresented in clinical trials (Ethun et al. 2017). Frailty is not defined by higher age alone, but includes patients that suffer from heterogeneous, significant co-morbidities or that have a higher susceptibility to severe adverse events and hospitalization (Ethun et al. 2017, Dale et al. 2023). Current treatment recommendations are mostly based on data from patients that are fit for intensive combination therapies, which, however, neglect this significant proportion of patients (Seymour et al. 2011).

Based on the data available, two different strategies may be applied to take significant comorbidities or frailty into account. Initial dose reduction of doublet chemotherapies to 80% showed comparable efficacy and quality of life compared to single-agent fluoropyrimidines (FPs) (Seymour et al. 2011). With the availability of targeted treatments, the addition of bevacizumab to oral FPs as alternative strategy was associated with prolongation of the progression-free survival (PFS) in frail patients (Cunningham et al. 2013, Cutsem et al. 2020). Nevertheless, innovations in targeted treatment such as the application of monoclonal antibodies directed against the epidermal growth factor receptor (EGFR) to *RAS* wild-type (WT) tumors were not considered by those trials. As shown by the randomized phase II PANDA trial, the addition of panitumumab to FOLFOX or FU/FA was equally effective but associated with less adverse events when combined with FU/FA (Lonardi et al. 2023). Data for the addition of EGFR-directed monoclonal antibodies to oral FP is, however, limited. One of the concerns was additive toxicities for capecitabine in combination with EGFR-antibodies, especially with regard to skin and gastrointestinal toxicities.

We therefore aimed to perform a systemic literature review of the evidence available for the combination of oral FP with EGFR-directed monoclonal antibodies in frail patients with *RAS* WT mCRC to assess efficacy and tolerability in first-line and later-line regimens.

## Methods

The systematic review was reported according to the PRISMA (Preferred Reporting Items for Systematic Reviews and Meta-Analyses checklist) strategy. The goal of this review was to evaluate data on the efficacy and safety for combining an oral FP with an EGFR-directed antibody as first-line treatment for *RAS* WT mCRC.

### Selection process

Title/abstracts were screened for eligibility followed by full-text review. The screening was based on the following inclusion criteria: trials phase II and phase III, primary studies analyzing patients with metastatic colorectal cancer treated with oral FP and EGFR-directed antibody. Exclusion criteria were defined as: other tumor entities, phase I trials, reviews, meta-analyses, retrospective analyses, single-center studies, no combination of just oral fluoropyrimidine and anti-EGFR-antibody.

Results were divided in treatment in first-line setting and later-line setting defined as all treatment from second line. All articles were published in English and did not need translation.

Data bases and date: A comprehensive search was conducted through PubMed database on November 26th, 2024, and Cochrane Library on November 29th, 2024. This analysis was expanded on both databases on August 1st, 2026. We applied the following search terms: (Trifluridine/Tipiracil OR Lonsurf OR TAS 102 OR capecitabine OR S-1 OR oral fluoropyrimidine) and (anti-EGFR-antibody OR panitumumab OR Vectibix OR cetuximab OR Erbitux) in metastatic colorectal cancer.

### Data collection

Data was extracted using a standardized excel-spread sheet.

### Data outcome

 The following outcomes were extracted from the trials analyzed: study characteristics, previous lines of treatment, 1st author, year of publication, country of publication, trial characteristics, number of patients included in the trial, median age, location of primary tumor, treatment and endpoints, adverse events.

### Risk of bias

The following domains were evaluated according to the RoB-2 framework for randomized trials. The following domains were analyzed: (1) bias due to the randomization process, (2) bias due to deviations from the intend interventions, (3) bias due to missing outcome data, (4) bias in measurement of the outcome, (5) bias in selection of the reported results. The domains were judged as “low risk,” “some concerns,” or “high risk”. For the non-randomized trials bias assessment was evaluated according to the ROBINS-I framework. The following domains were analyzed: (1) bias due to confounding, (2) bias in selection of participants into the study, (3) bias in classification of the interventions, (4) bias due to deviations of intended interventions, (5) bias due to missing data, (6) bias in measurement of outcome, (7) bias in selection of the reported results. The domains were judged as “low risk of bias,” “moderate risk of bias,” or “serious risk of bias”, “critical risk of bias”, “no information”. The risk of bias assessment was performed by the first author.

### Effect measures 

Effect was measured in difference between overall survival (OS), progression-free survival (PFS), disease control rate (DCR) and objective response rate (ORR) as well as comparison of adverse events.

**Fig. 1:**
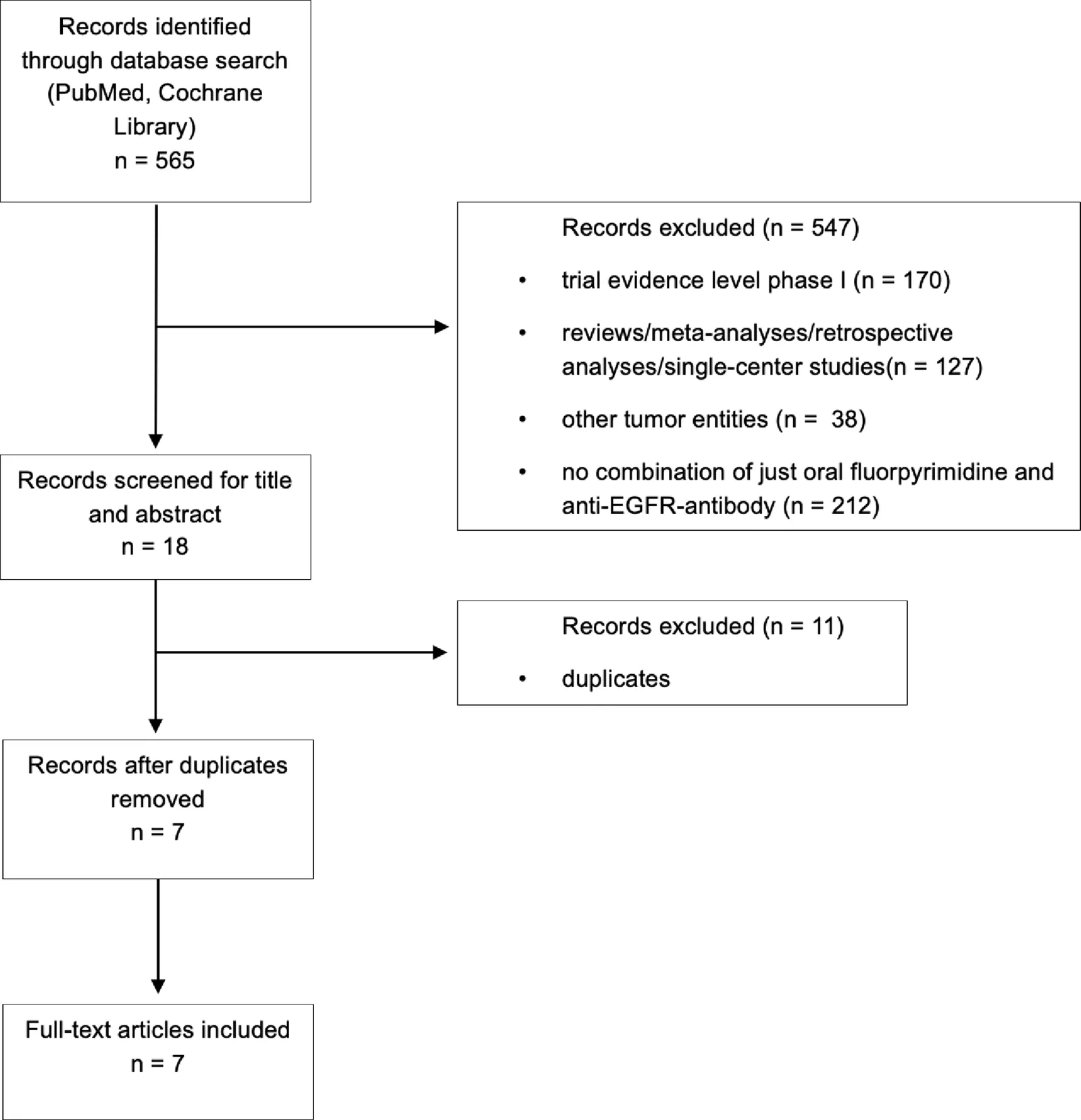
Results PRISMA flow diagram

## Results

### Selection of studies

With the dedicated search criteria, we identified 565 trials through PubMed and Cochrane Library, of which 547 records were excluded (Fig. 1). 18 abstracts were screened, of which 11 duplicates were excluded. Finally, 7 publications of 6 studies were selected for full-text review and data extraction (Fig. 1).

### First-line treatment

A total of 40 elderly untreated patients received an oral FP in combination with an anti-EGFR antibody in two phase II studies (capecitabine plus cetuximab (CC): *n* = 13; capecitabine plus panitumumab (CP): *n* = 27).

Both trials showed similar median PFS (CC: 7.2 months, 95% CI 4.6–15.5 months; CP: 7.5 months, 95% CI 4.4–10.4 months). OS of patients receiving CC was 17.1 months (95% CI not defined) and 23.7 months (95% CI: 7.4–27.5 months) for treatment with CP, respectively. The DCR of CC and CP were 69.2% and 70.4%, respectively, and ORR were 30.8% and 44.4% (Kienle et al. 2019, Méndez Méndez et al. 2020).

The rate of grade ≥ 3 treatment-emergent AE was not reported for CC and was 63% in patients treated with CP. While rash grade ≥ 3 was not observed in patients treated with CC, CP was associated with grade ≥ 3 rash in 18.5% of patients (Kienle et al. 2019, Méndez Méndez et al. 2020).

The study characteristics are displayed in Tables 1, 2.

**Table 1:** Risk of bias assessment

Trial	Tool	Domain 1	Domain 2	Domain 3	Domain 4	Domain 5	Domain 6	Domain 7
VELO [9, 12]	RoB-2	Low risk	Low risk	Low risk	Some concerns	Low risk	–	–
SAKK41/10 [7]	RoB-2	Some concerns	Low risk	Some concerns	Some concerns	Some concerns	–	–
PANEL [8]	ROBINS-I	Serious risk	Low risk	Low risk	Moderate risk	Moderate risk	Moderate risk	Moderate risk
APOLLON [11]	ROBINS-I	Serious risk	Low risk	Low risk	Low risk	Moderate risk	Moderate risk	Low risk
KSCC0901 [13]	ROBINS-I	Serious risk	Low risk	Low risk	Low risk	Moderate risk	Moderate risk	Low risk
WJOG8916G [10]	ROBINS-I	Serious risk	Low risk	Low risk	Low risk	Low risk	Low risk	Low risk

**Table 2:** Characteristics of the studies (first-line)

1st author, year of publication, country of publication	Study characteristics	Total no. of patients	Median age (years)	Location of primary tumor no. (%)	Treatment	Primary endpoint
Kienle et al. 2019 Switzerland	RCT, Multicenter, Phase II SAKK 41/10 study *Stopped prematurely due to slow acquisition*	24	80	**A** Left: 5 (38.5) Right: 3 (23.1) Rectum: 5 (38.5) **B** Left: 6 (54.5) Right: 3 (27.3) Rectum: 2 (18.2)	Cmab 500mg/m^2^ d1 qw2 + capecitabine 1000mg/m^2^ 2x/day d1-14 qw3 vs. Cmab 500mg/m^2^ d1 qw2	PFS at 12 weeks
Méndez et al. 2020 Spain	Single-arm, phase II PANEL study	27	78	Colon: 24 (88.9) Rectum: 3 (11.1)	Pmab 9 mg/kg d1 + capecitabine 850mg/m^2^ 2x/day d1-14 qw3	ORR

RCT = randomized controlled trial, arm A: combination arm, arm B: monotherapy arm, Cmab = cetuximab, Pmab = panitumumab, FTD/TPI = trifluridine/tipiracil, mos = months, mPFS = median progression-free survival, mOS = median overall survival, DCR: disease control rate, ORR = objective response rate, n.a. = not available, AEs = adverse events

### Later-line treatment

The third-line treatment of FPs and EGFR-directed antibodies was investigated in four phase II studies (FTD/TPI plus panitumumab (*n* = 2) or cetuximab (*n* = 1), S1 plus cetuximab (*n* = 1)) in younger, but heavily pre-treated patients with refractory mCRC. In total, 211 patients were treated (FTD/TPI plus panitumumab: *n* = 118; FTD/TPI plus cetuximab: *n* = 56; S-1 plus cetuximab: *n* = 37). Two of these studies were designed as anti-EGFR re-challenge concepts (i.e., the re-introduction of anti-EGFR treatment after formal progression and switch of treatment strategy) (Napolitano et al. 2023, Izawa et al. 2023).

The median PFS was longest for FTD/TPI plus panitumumab (APOLLON: 5.8 months, 95% CI 4.5–6.5; VELO: 4.0 months, 95% CI: 2.8–5.3), followed by S-1 plus cetuximab (5.5 months, 90% CI: 4.4–5.7) and re-challenge with FTD/TPI with cetuximab (2.4 months, 95% CI: 2.1–3.7) (Napolitano et al. 2023, Izawa et al. 2023, Kato et al. 2021, Napolitano et al. 2023, Takahashi et al. 2016).

In terms of OS, comparable results were observed for FTD/TPI plus panitumumab (APOLLON: 14.1 months, 95% CI: 12.2–19.3; VELO: 13.1 months, 95% CI: 9.5–16.7) and S-1 plus cetuximab (13.5 months, 95% CI: 8.5–16.5) (Napolitano et al. 2023, Kato et al. 2021, Napolitano et al. 2023, Takahashi et al. 2016). The combination of FTD/TPI plus cetuximab as re-challenge resulted, however, in numerical shorter OS (9.8 months, 95% CI: 7.4–12.2) (Izawa et al. 2023).

The DCR were higher for FTD/TPI plus panitumumab (APOLLON: 81.5%, 95% CI: 68.6–90.8; VELO: 74.2%, 95% CI not reported) and S-1 plus cetuximab (73%, 95% CI not reported) compared to FTD/TPI with cetuximab re-challenge (54%, 80% CI: 44.0% – 63.0%) (Napolitano et al. 2023, Kato et al. 2021, Napolitano et al. 2023, Takahashi et al. 2016).

ORR were highest in patients not previously treated with anti-EGFR agents (FTD/TPI plus panitumumab (APOLLON): 37.0%, 95% CI 24.3–51.3; S-1 plus cetuximab: 29.7%, 95% CI: 15.9% – 47.0%), followed by re-challenge with FTD/TPI plus panitumumab (VELO: 9.7%, 95% CI not reported) or cetuximab (3.6%, 95% CI 0.4% – 12%) (Napolitano et al. 2023, Izawa et al. 2023, Kato et al. 2021, Napolitano et al. 2023, Takahashi et al. 2016).

The rate of grade ≥ 3 treatment-emergent AEs was 51.6% (VELO) to 76.4% (APOLLON) for the combination of FTD/TPI plus panitumumab. Cumulative rates were not reported for FTD/TPI plus cetuximab re-challenge or S-1 plus cetuximab (Takahashi et al. 2016). Across all studies, the most frequent hematological grade ≥ 3 adverse event was neutropenia, and the most frequent non-hematological grade ≥ 3 adverse events were skin rash and/or acneiform dermatitis (Napolitano et al. 2023, Izawa et al. 2023, Kato et al. 2021, Napolitano et al. 2023, Takahashi et al. 2016).

The study characteristics are displayed in Tables 3 and 4.

**Table 3:** Characteristics of the studies (later-lines)

1st author, year of publication, country of publication	Study characteristics	Total no. of patients	Median age (years)	Location of primary tumor no. (%)	Treatment	Primary endpoint
Kato et al. 2021 Japan	Single-arm, multicenter, phase II APOLLON study	56	63	Left: 48 (87.3) Right: 7 (12.7)	Pmab 6 mg/kg qw2 + FTD/TPI 35 mg/m2 2x/day; days 1–5 and 8–12 qw4	PFS at 6 months
Napolitano et al. 2023 Italy	RCT, phase II VELO study	62	65	n.a.	Pmab 6 mg/kg d1 and d15 qw2 of a 28-day cycle + FTD/TPI 35 mg/m^2^ 2x/day d1-5, d8-12, followed by a 14-day rest period vs. FTD/TPI 35 mg/m^2^ 2x/day d1-5, d8-12, followed by a 14-day rest period	PFS
Napolitano et al. 2023 Italy	RCT, phase II, Subgroup analysis VELO study	62	65	n.a.	Pmab 6 mg/kg d1 and d15 qw2 of a 28-day cycle + FTD/TPI 35 mg/m^2^ 2x/day d1-5, d8-12, followed by a 14-day rest period	OS

RCT = randomized controlled trial, arm A: combination arm, arm B: monotherapy arm, Cmab = cetuximab, Pmab = panitumumab, FTD/TPI = trifluridine/tipiracil, mos = months, mPFS = median progression-free survival, mOS = median overall survival, DCR: disease control rate, ORR = objective response rate, n.a. = not available, AEs = adverse events

**Table 4:** Characteristics of the studies with anti-EGFR rechallenge (later-lines)

1st author, year of publication, country of publication	Study characteristics	Total no. of patients	Median age (years)	Location of primary tumor no. (%)	Treatment	Primary endpoint
Takahashi et al. 2016 Japan	Single-arm, multicenter, phase II KSCC 0901 study	37	64	n.a.	Cmab initial dose 400 mg/m^2^, followed by weekly infusion at 250 mg/m^2^. S-1 80 mg/m^2^ 2x/day for 28 days followed by a 14-day rest period	PFS
Izawa et al. 2023 Japan	Single-arm, multicenter, phase II WJOG8916G study	56	60	Left: 59 (91 Right: 1 (9) Rectum: /	FTD/TPI 35 mg/m^2^ twice daily on days 1–5 and 8–12 + Cmab initially 400 mg/m^2^, followed by weekly 250 mg/m^2^ qw4	DCR

*Legend*: RCT = randomized controlled trial, arm A: combination arm, arm B: monotherapy arm, Cmab = cetuximab, Pmab = panitumumab, FTD/TPI = trifluridine/tipiracil, mos = months, mPFS = median progression-free survival, mOS = median overall survival, DCR: disease control rate, ORR = objective response rate, n.a. = not available, AEs = adverse events

## Discussion

In this review, we evaluated the treatment effects of an oral FP in combination with EGFR-directed antibodies in frail patients with mCRC in first and later lines. According to our definition, frailty included advanced age, significant comorbidities, and/or extensive prior treatment with refractory disease.

Although frail patients with colorectal cancer represent the most common population in daily oncology practice, the term “frailty” is not uniformly defined in clinical studies, which makes comparative evaluation difficult overall. The patient’s age is of crucial importance, but so are comorbidities and ECOG performance status. It would therefore be incorrect to define a contraindication for combination chemotherapy based solely on age, and besides the SAKK 41/10 and PANEL trials, the median age in all studies was below 70 years. Nevertheless, the evidence in younger cohorts shows promising efficacy and adverse event data, which would also be of interest for frail patients. This systematic review therefore included all studies in which patients were treated with oral FP and anti-EGFR antibodies in order to avoid the deliberate exclusion of patients. However, this also led to the identification of re-challenge studies, whose effectiveness after progression to a previous targeted therapy is understandably lower than that of a regimen in patients without documented progression. A potential selection bias of the studies could not be excluded.

With reduced doublet chemotherapy and FP monotherapy, there are essentially two strategies available for treating frail patients. The MRC FOCUS2 trial evaluated the feasibility of initially dose-reduced regimens at 80% of the original dose with a median PFS of 5.8 months and OS of 10.7 months for FOLFOX, and grade 3 or higher adverse events of 33% (Seymour et al. 2011). The study was conducted in the pre-antibody era, so that a comparison of the efficacy data to the FP plus anti-EGFR antibody strategies is not directly applicable, not least because of the lack of head-to-head comparisons in clinical studies. There are four arguments however that favor the use of FP monotherapy over the use of a reduced doublet. First, the introduction of anti-EGFR agents to doublet chemotherapies in patients with *RAS* WT mCRC led to an absolute benefit of up to 2.2 months for PFS and 5.8 months for OS when compared to sole first-line chemotherapy (Douillard et al. 2013, Cutsem et al. 2009). In this context, based on the data presented, monotherapy with FP and anti-EGFR antibody would probably not be inferior to the dose-reduced doublet with anti-EGFR antibody, apart from the re-challenge studies. Secondly, it cannot be ruled out that chemotherapy, which has already been reduced in dose at the start of treatment in frail patients, may need to be reduced further during the course of therapy, which may also further reduce the described effectiveness. Thirdly, the recently published PANDA study showed that there was no additional benefit for frail patients from the use of oxaliplatin in combination with 5-fluorouracil, folinic acid, and panitumumab (Lonardi et al. 2023). Fourthly, even though there is no direct comparison between the efficacy and tolerability of oral FPs vs. intravenous FPs in combination with anti-EGFR antibodies, the efficacy of oral fluoropyrimidines as capecitabine to infusional 5-fluorouracil regimens as monotherapy or in combination with oxaliplatin and/or bevacizumab was considered non-inferior in previous trials (Hoff et al. 2001, Ducreux et al. 2011, Ducreux et al. 2013, Díaz-Rubio et al. 2007), albeit with slightly higher risks for grade 3 adverse events when using oral FP (Seymour et al. 2011). From the patients’ perspective however, regarding quality of life, the simplified application of an oral therapy compared to an infusion lasting several hours and a pump over 48 h would be associated with fewer visits to the practitioner overall. Moreover, patients would not routinely require a port system, as EGFR-directed antibodies can be administered via peripheral venous catheters, reducing the risk of port-associated complications.

By contrast, the ESMO guideline and an expert panel recommendation stated limited efficacy of capecitabine and combinations with irinotecan or oxaliplatin together with anti-EGFR agents (Committee, E.G et al. 2026, Maughan et al. 2011). The available evidence is driven by capecitabine-based studies, whereas other fluoropyrimidines have not yet been sufficiently studied yet. Other fluoropyrimidines, such as FTD/TPI, may offer the potential for improved tolerability when used in combination with monoclonal antibodies: a direct comparison of FTD/TPI and capecitabine combined with bevacizumab indicated higher rates of hematological, but lower rates of limiting gastrointestinal toxicities and hand-foot-syndrome with regard to FTD/TPI (Cutsem et al. 2020, André et al. 2023). This could potentially improve the tolerability of FP monotherapies when used in combination with anti-EGFR antibodies.

This systematic review had several limitations. The studies included had small sample sizes and the study populations enrolled were heterogeneous, especially for patients treated in first-line studies (*n* = 40), making generalization difficult. However, this analysis aimed to extrapolate the efficacy and safety data to frail patients, who are still underrepresented in the available trials. Moreover, there is no unified definition of frailty, which is why its definition might vary between individual studies. The number of trials in the later-line setting is predominant, and subgroup analyses were included. Two trials in the later-line setting were re-challenge concepts differing from SOC, however, suggesting a potential benefit for a molecular defined subgroup (Napolitano et al. 2023, Izawa et al. 2023). Also, tumor sidedness should be incorporated more explicitly into future research since the existing evidence for anti-EGFR treatment benefit is strongest in *RAS* WT/*BRAF* WT/left-sided mCRC (Committee, E.G et al. 2026).Additionally, the emergence of subcutaneously administered antibodies provides an opportunity to evaluate this combination with oral FPs. This approach may improve convenience and reduce time spent in the outpatient clinic. For example, amivantamab, an EGFR-MET bispecific antibody, has shown promising activity in chemo-refractory *RAS* WT/*BRAF* WT mCRC and feasibility for subcutaneous application in solid tumors (Oberstein et al. 2026, Burtness et al. 2026). These developments expand the range of potential treatment strategies. In general, prospective, ideally randomized, studies are necessary to further analyze the safety of oral FP therapy in combination with anti-EGFR therapy. The ongoing FIRE-8 (AIO KRK-0519) study (NCT05007132), which compares FTD/TPI plus panitumumab in comparison to FTD/TPI plus bevacizumab in frail patients with RAS WT mCRC and contraindication to doublet chemotherapy, was therefore designed to clarify whether this population benefits from this combination in first-line setting.

## Conclusion

We performed this systematic review in order to collect the available evidence of oral FPs combined with anti-EGFR treatment with a focus on frail patients. While capecitabine should not be used together with anti-EGFR antibodies, data for FTD/TPI in combination with an EGFR-directed antibody is still scarce and requires further prospective investigation of the frail population in clinical trials.

## Data Availability

No datasets were generated or analysed during the current study.
